# Cell-Surface and Nuclear Receptors in the Colon as Targets for Bacterial Metabolites and Its Relevance to Colon Health

**DOI:** 10.3390/nu9080856

**Published:** 2017-08-10

**Authors:** Sathish Sivaprakasam, Yangzom D. Bhutia, Sabarish Ramachandran, Vadivel Ganapathy

**Affiliations:** Department of Cell Biology and Biochemistry, Texas Tech University Health Sciences Center, Lubbock, TX 79430, USA; sathish.sivaprakasam@ttuhsc.edu (S.S.); yangzom.d.bhutia@ttuhsc.edu (Y.D.B.); s.ramachandran@ttuhsc.edu (S.R.)

**Keywords:** colonic bacteria, symbiotic relationship, bacterial metabolites, molecular targets, cell-surface receptors, nuclear receptors, immune tolerance, colitis, colon cancer

## Abstract

The symbiotic co-habitation of bacteria in the host colon is mutually beneficial to both partners. While the host provides the place and food for the bacteria to colonize and live, the bacteria in turn help the host in energy and nutritional homeostasis, development and maturation of the mucosal immune system, and protection against inflammation and carcinogenesis. In this review, we highlight the molecular mediators of the effective communication between the bacteria and the host, focusing on selective metabolites from the bacteria that serve as messengers to the host by acting through selective receptors in the host colon. These bacterial metabolites include the short-chain fatty acids acetate, propionate, and butyrate, the tryptophan degradation products indole-3-aldehyde, indole-3-acetic, acid and indole-3-propionic acid, and derivatives of endogenous bile acids. The targets for these bacterial products in the host include the cell-surface G-protein-coupled receptors GPR41, GPR43, and GPR109A and the nuclear receptors aryl hydrocarbon receptor (AhR), pregnane X receptor (PXR), and farnesoid X receptor (FXR). The chemical communication between these bacterial metabolite messengers and the host targets collectively has the ability to impact metabolism, gene expression, and epigenetics in colonic epithelial cells as well as in mucosal immune cells. The end result, for the most part, is the maintenance of optimal colonic health.

## 1. Introduction

Over millions of years, humans have co-evolved with microorganisms through co-habitation. Adult human harbors microflora that is equal or even greater than their human cells in number [1,2]. These microorganisms colonize different parts of the body that are exposed to external environment, which include skin, oral cavity, airway lumen, intestinal tract, and vagina. Among these, the colon is the site where the microorganisms are most abundant, with bacteria as the principal component. There are 800–1000 different species of colonic bacteria under normal physiological conditions, but the presence or absence of specific strains and the relative abundance of any given strain might vary from individual to individual, primarily influenced by the environmental factors such as the diet, air and water quality, medications, and mode of delivery (vaginal or Cesarean section) [3]. Because of the impact of multiple variables, the colonic bacteria might not remain the same even in a given individual over a long period of time. Despite these interpersonal variations, colonic bacteria are in general dominated by two phyla: Bacteroidetes and Firmicutes [4,5]. Normal microflora in the colon elicit significant influence on the host, in the colon and in other organs. Recent studies have implicated colonic bacteria in brain function (gut-brain axis), liver function (gut-liver axis), mucosal and systemic immune function, diabetes (type 1 and type 2) (gut-pancreas axis), nutrition and obesity, and cardiovascular diseases [6,7,8,9,10]. This broad spectrum of biological impact of colonic bacteria on the host is possible because of active communication between the two co-habitants. The impact of colonic bacteria on the host is not limited locally to the colon because of the changes the bacteria bring in terms of gene expression and biological function in colonic epithelial cells as well as mucosal immune cells. The colon releases a wide variety of biologically active molecules into circulation in response to changes in the density and phylogenic makeup of the bacterial population; these active molecules include hormones and cytokines that impact on the functions of distant organs including the liver, pancreas, and brain. Similarly, mucosal immune cells are also influenced in their behavior and function in response to colonic bacteria, which then travel to distant sites to modulate immune function and disease processes [11,12]. Furthermore, colonic bacteria themselves elaborate different classes of metabolites, which enter the portal and systemic circulations to regulate the biology of cells and tissues at distant sites through specific molecular targets that include enzymes and receptors [10,13,14]. 

Because of its close proximity to the bacteria, the colon is the most influenced organ as the result of this co-habitation. The presence of bacteria in the colon is absolutely necessary for optimal colonic health; normal bacteria in the colon offer protection against inflammation and cancer. As such, the beneficial effects of colonic bacteria are undeniable. Any imbalance in the normal density and phylogenetic composition in colonic bacteria, generally referred to as dysbiosis, is detrimental to colonic health [15,16,17,18]. Inflammatory bowel diseases (IBD) are chronic inflammatory disorders of the intestinal tract that develop as a result of deregulation of the immune response toward the intestinal/colonic bacteria. In particular, there is a marked decrease in the colonization of Firmicutes and Bacteroidetes in the gut microbiota in association with IBD. Crohn’s disease and ulcerative colitis are the two major types of IBD, which are distinct diseases with different histological features and etiology that involve different immune cell types. Animal studies have shown that neither of these diseases develops under germ-free conditions, indicating that the presence of bacteria is obligatory for the disease process [19,20]. The findings that normal bacteria are essential for colonic health and that IBD does not develop in the absence of bacteria are not necessarily contradictory. The epithelial cells of the intestine/colon form an effective barrier between the bacteria and the host, and the communication between the two co-habitants occurs solely via chemical messengers to make sure that the mucosal immune system is not overly active in response to the bacterial antigens. However, if the barrier function is compromised for any reason, the ability of the mucosal immune system to tolerate the bacteria is at risk [21,22,23]. What causes the breakdown of the barrier in IBD is not completely understood, but changes in the normal bacterial phylogenetic composition are believed to be major determinants in the process. When the phylogenetic composition (i.e., dysbiosis) is altered in the colon, the chemical communication between the bacteria and the host also undergoes changes, thus initiating the inflammatory disease process. This phenomenon is also relevant to carcinogenesis in the colon because chronic inflammation as occurs in ulcerative colitis increases the risk of colorectal cancer [24]. 

## 2. Relationship between Microbiota and the Host: Parasitism, Commensalism, or Mutualism?

The relationship between the host and the microbiota could fall into three categories—parasitism, commensalism, and mutualism—depending on the specifics of the interaction. In parasitism, one of the co-habitants gets the benefits, while the other is harmed. In commensalism, one of the co-habitants reaps the benefits, while the co-habitation is neither beneficial nor harmful to the other partner. In mutualism, both partners get the benefits from the co-existence. The relationship between the host and the colonic bacteria falls under the third category, mutualism. The benefits to the bacteria as a result of this co-habitation include the availability of the colonic lumen for colonization and proliferation and provision of the nutrients by the host necessary for survival and growth. The host also benefits from the relationship [25,26,27]. Colonic bacteria synthesize a variety of vitamins (folic acid, biotin, vitamin K, etc.) that are made available to the host. The principal fermentation products of bacterial metabolism, called short-chain fatty acids, that are present in normal colonic lumen at concentrations as high as 100 mM serve as preferential metabolic fuel to colonic epithelial cells [28]. These short-chain fatty acids also function as signaling molecules eliciting a broad range of biological effects in colonic epithelium by targeting enzymes such histone deacetylases (HDACs), cell-surface receptors such as GPR109A and GPR43, and nuclear receptors such as AhR, PXR, FXR, and peroxisome proliferator-activated receptors (PPARs) [29]. The end result of this mutually beneficial co-habitation is a symbiotic relationship between the two partners. Notwithstanding this overwhelming evidence for mutualism between the host and colonic bacteria, too many investigators and published reports still continue to describe the relationship between the host and the bacteria as commensalism and the bacteria as commensals [30,31,32]. Surprisingly, the terms “commensalism” and “commensals” appear even in publications that provide data in support of the mutually beneficial relationship between the host and the colonic bacteria [25,30,31,32].

## 3. Bacterial Dysbiosis as a Cause of Colonic Diseases

Dysbiosis in the colon is a critical determinant in the pathogenesis of IBD. Research on colitis induction in germ-free mice with introduction of specific strains of bacteria has shown that microbial dysbiosis, rather than genetic factors, is the major factor for IBD pathogenesis. Many genetically-engineered mouse models that develop spontaneous colitis in the presence of bacteria in the colon are resistant to colitis when raised under germ-free conditions [33,34]. This suggests that the presence of bacteria in the intestinal tract is a critical requirement for IBD development. Most animal models of colitis show altered bacterial composition in the colon, providing evidence of dysbiosis [16,35]. However, the link between dysbiosis and IBD is only associative, and the cause-effect relationship has not yet been established beyond doubt. Nonetheless, administration of probiotics to patients with colonic inflammation has beneficial effects in terms of slowing or preventing progression of the disease process [36,37,38]. As these probiotics tend to favor colonization of normal bacterial flora and suppress the growth of pathogenic bacteria in the colon, the preventive effects of probiotics suggest effective reversal of the disease-associated dysbiosis and may actually point to a mechanistic model in which the dysbiosis is most likely the cause, rather than the result, of colonic inflammation [37,39,40].

## 4. Bacterial Metabolites with Impact on the Host: Relevance to Colitis and Colon Cancer

The communication between colonic bacteria and the host could be contact-dependent as well as contact-independent. The phylogenetic composition of the microflora in colonic lumen is not exactly the same as that of the microflora on the surface of the colonic epithelium. Which bacterial strains adhere to the colonic epithelial cells depends on multiple factors such as the presence or absence of cell-surface receptors for a given strain, ability of bacterial strains to traverse the thick mucin present on the luminal surface of the epithelial cell layer, as well as susceptibility of the bacterial strains to the bactericidal and bacteriostatic actions of the various molecules secreted by the epithelial cells and mucosal immune cells. The physical contact followed by insertion of specific bacterial proteins into the epithelial cell membrane could elicit signaling changes in the epithelium as a means of communication. Another mode of communication, probably more prevalent than the former, is through metabolites released into the lumen from live bacteria or from dead bacterial cells. The colon provides a favorable environment for anaerobic microbial growth, resulting in fermentation by resident bacteria of dietary constituents that reach the colon. The microbiota population in the colon is highly variable depending on the intestinal physiology, substrate availability, oxygen tension, and pH [28]. As a result, the microorganisms present in the colon are not the same all through the length of the large intestine. The phylogenetic composition of the bacteria is significantly different between the proximal colon and the distal colon [41]. As the metabolites elaborated by these bacteria depend on the repertoire of metabolic pathways in a given strain of the bacteria, different regions of the colon are exposed to different bacterial metabolites and hence respond differently. Furthermore, the phylogenetic composition of the colonic bacteria varies from individual to individual and also in the same individual in health and disease; this again impacts the biological response of the host colon to the presence of bacteria that could be significantly different on an individual basis and also depend on the health status of the individual.

Among these bacterial metabolites responsible for communication between the bacteria and the host, short-chain fatty acids (SCFAs) have received the most attention [42,43,44,45]. SCFAs are organic acids consisting of 2–4 carbon atoms. The principal components of SCFAs are acetate (C2), propionate (C3), and butyrate (C4). These fatty acids arise in the colon by bacterial fermentation of carbohydrates that reach the colon either in the form of dietary fiber or due to ineffective digestion and absorption in the small intestine. The relative ratio of acetate, propionate, and butyrate in colon is 6:3:1, and the total concentration of these three SCFAs in colonic lumen is in the range of 50–100 mM. SCFAs serve as an important energy source for colonocytes. These fatty acids enter the colonic epithelium across the lumen–facing apical membrane by diffusion, anion-exchange, H^+^/SCFA^−^ symport, and Na^+^/SCFA^−^ symport [46,47,48,49]. The relative contributions of each of these entry mechanisms might vary depending on the luminal concentrations of SCFAs because of the marked differences in the substrate affinities for the transport processes. Under physiological conditions with luminal concentrations of SCFAs in the range of 50–100 mM, diffusion is probably the major entry mechanism. When the concentrations decrease due to changes in the dietary intake of fibers or due to dysbiosis under disease conditions, the carrier-mediated entry mechanisms probably play the major role. Irrespective of the entry mechanism, most of the SCFAs generated by the bacteria are absorbed in the colon, with only a minimal amount excreted in feces. The biological effects of SCFAs in the colon are not restricted to their role as energy substrates for the epithelial cells. These bacterial metabolites promote water and electrolyte absorption in the colon, thus providing protection against potential diarrheagenic diseases [48]. They also modulate the mucosal immune system by helping the development of a tolerant environment to facilitate the co-habitation of the bacteria and the host [50,51,52]. They aid in the maintenance of the mucosal barrier, thus preventing the direct exposure of systemic organs to colonic bacteria. SCFAs also suppress colonic inflammation and carcinogenesis [43,46,53,54,55,56].

As the cell-surface receptors for SCFAs are located on the lumen-facing apical membrane of colonic epithelial cells (see below), the luminal concentrations of these agonists are physiologically relevant. SCFAs are low-affinity agonists for these receptors, and the normal luminal concentrations of these bacterial metabolites are in the millimolar levels, sufficient to activate these receptors from the luminal side. However, some of the molecular targets for these metabolites are either inside the cells (e.g., HDACs) or on the surface of the immune cells located in the lamina propria. Therefore, concentrations of these metabolites inside the colonic epithelial cells and in the lamina propria are relevant to impact these molecular targets. The intracellular target HDAC is inhibited by butyrate and propionate at low micromolar concentrations. There are effective transport systems for SCFAs in the apical membrane of colonic epithelial cells (e.g., proton-coupled and sodium-coupled monocarboxylate transporters) [47], thus making it very likely for these SCFAs to reach intracellular levels sufficient to inhibit HDACs. Even though the luminal concentrations of SCFAs are in the millimolar range, it is unlikely that they reach lamina propria at significant levels to activate the cell-surface receptors present on the mucosal immune cells. These metabolites are present only at micromolar levels in the portal blood [57], indicating that they undergo robust metabolism inside the colonic epithelial cells. This raises the question as to the physiological relevance of these bacterial metabolites to the activation of the cell-surface SCFA receptors in immune cells located in the lamina propria. With regard to this issue, it is important to note that colonic epithelial cells are highly ketogenic; they use acetate and butyrate to generate the ketone body β-hydroxybutyrate [58]. This ketone body is released from the cells into portal blood. As β-hydroxybutyrate is 3–4 times more potent than butyrate in activating its receptor GPR109A, it can be speculated that the colon-derived ketone body is most likely involved in the activation of the SCFA receptor in mucosal immune cells. 

Lactate is another bacterial metabolite that is important to colonic health but has received much less attention than the SCFAs. Lactobacilli that generate lactate as a fermentation product form a significant constituent of normal bacterial flora in the colon. As such, in addition to the dietary sources of lactate from yogurt and other dairy products, bacterial metabolism constitutes an important source of lactate in the colonic lumen [36,59].

Bacterial metabolites arising from protein catabolism are receiving increasing attention in recent years, particularly the metabolites resulting from tryptophan degradation [60,61]. This includes indole, indole-3-aldehyde, indole-3-acetic acid, and indole-3-propionic acid. These metabolites also elicit beneficial effects on the host colon; they suppress inflammation and carcinogenesis [14,62]. Another important class of bacterial metabolites that impact on colonic health is the secondary bile acids [63]. Liver synthesizes two different bile acids, namely cholic acid and chenodeoxycholic acid, which are called primary bile acids. These bile acids are secreted into the bile and reach the small intestine to facilitate the digestion and absorption of dietary fat. Almost 95% of these bile acids are reabsorbed in the ileum to undergo entero-hepatic circulation. The remaining bile acids reach the colon where the resident bacteria metabolize them into secondary bile acids (deoxycholic acid and lithocholic acid). The bacteria-generated secondary bile acids get absorbed in the colon and enter the entero-hepatic circulation similar to the primary bile acids produced by the liver. Colonic bacteria also act on dietary lipids, generating metabolites such as conjugated linoleic and linolenic acids and trimethylamine [64,65,66]. All these molecules have marked biological effects on the host colon. In addition to these metabolites, the bacterial cell-wall components such as lipopolysaccharide, peptidoglycans, and polysaccharide A also serve signaling molecules in the colon. 

## 5. Molecular Targets for Bacterial Metabolites in the Host

Even though the beneficial effects of colonic bacteria and their metabolites on the host have been recognized for a long time, it was not until recently that we began to understand the molecular mechanisms that underlie this important biological phenomenon. This was true even in the case of SCFAs for which there is a long history of investigations focusing on the role of these bacterial metabolites as the molecular link between colonic bacteria and the host. Inhibition of histone deacetylases (HDACs) by the SCFA butyrate is an exception; HDAC is probably the first molecular target discovered for bacterial metabolites in the colon to provide a mechanistic insight into the communication between colonic bacteria and the host [67]. HDACs are enzymes that modulate the epigenetics of the target cells, including the colonic epithelium. Butyrate is an inhibitor of HDAC1 and HDAC3, which belong to class I HDACs [68,69]. Propionate also possesses this inhibitory effect [68,69]. There is overwhelming evidence in the literature for overexpression of HDACs in cancer and for the therapeutic rationale and efficacy of HDAC inhibitors in cancer treatment [70,71]. As such, the biologic phenomenon of protection against colon carcinogenesis by SCFAs can be explained, at least in part, by the functions of butyrate and propionate as inhibitors of HDACs. This also provided a mechanistic explanation for the tumor-suppressive function of the Na^+^-coupled SCFA transporter SMCT1 (SLC5A8) in the colon [46]. HDACs being intracellular enzymes, entry of SCFAs from the lumen into colonic epithelial cells is a prerequisite for these bacterial metabolites to reach their molecular targets inside the cells. As inhibition of HDACs causes tumor suppression, it is understandable why the transporter that facilitates this process functions as a tumor suppressor.

In the past decade, we have witnessed a burgeoning of literature describing identification of new and novel molecular targets in the colon for bacterial metabolites [29,50,51,60,61]. These new discoveries have expanded our current understanding and knowledge of the molecular mechanisms as the basis of effective communication between the colonic bacteria and the host. The newly identified molecular targets include the cell-surface G-protein-coupled receptors GPR109A, GPR43, GPR41, GPR 81, and GPR91, and the nuclear receptors AhR (aryl hydrocarbon receptor), PXR (pregnane X receptor), and FXR (farnesoid X receptor). Table 1 lists the bacterial metabolites and the corresponding endogenous metabolites that function as agonists for each of these molecular targets. There are additional molecular targets for bacterial metabolites such as the receptors for bacterial cell-wall components (TLR4 for lipopolysaccharide, NOD1/NOD2 for peptidoglycans, and TLR2 for polysaccharide A) and the receptors for lipid metabolites from bacteria (TAAR5 for trimethylamine and PPARs for conjugated linoleic and linolenic acids) [29]. The present review however will focus on the cell-surface G-protein-coupled receptors GPR109A, GPR43, GPR41, GPR81, and GPR91, and the nuclear receptors AhR, PXR, and FXR. 

## 6. G-Protein-Coupled Receptors for Bacterial Metabolites

G-protein-coupled receptors are seven transmembrane domain–containing proteins involved in cellular signaling; most of them are expressed on the cell surface to interact with extracellular signals and transmit the signals into the cells via second messengers. The human genome encodes ~700 G-protein-coupled receptors. Even though physiological agonists have been identified for many of these receptors, a significant number of them still remain as orphan receptors with no information on the identity of their agonists. In the last decade, however, a distinct class of these “orphan” receptors has been found to be activated by normal physiological metabolites, which were never considered as signaling molecules; these include long-chain fatty acids, the ketone body β-hydroxybutyrate, the ubiquitous metabolite lactate, and the citric-acid-cycle intermediate succinate. Many of these metabolites are also found in the bacterial kingdom and hence are present in colonic lumen; consequently, if these receptors are expressed on the lumen-facing apical membrane of colonic epithelial cells, bacterial metabolites serve as the agonists for these receptors rather than the metabolites arising from the host metabolism. Examples of this category include GPR81, GPR91, GPR43, and GPR41 (Table 1). In some cases, the endogenous agonists for the receptors in non-colonic tissues are different from the bacterial metabolites, which activate the same receptors in the colon; in these cases, metabolites from bacteria that are structurally similar to endogenous agonists “hijack” the receptors for bacteria-host communication. Examples of this category include GPR109A, AhR, PXR, and FXR (Table 1).

## 7. GPR109A: Expression and Function in the Colon and the Mucosal Immune System

GPR109A was originally identified as a high-affinity receptor for the B-complex vitamin niacin (nicotinic acid) [87,88,89]. This provided a molecular mechanism for the niacin-induced correction of dyslipidemia because activation of the receptor in adipocytes inactivates the hormone-sensitive lipase and inhibits lipolysis. However, niacin is only a pharmacological agonist as the affinity of the receptor for niacin (~1 μM) is such that plasma concentrations of niacin under normal physiological conditions (~0.1 μM) are not sufficient to activate the receptor. Subsequently, β-hydroxybutyrate, the major ketone body in blood, was identified as the physiological agonist for the receptor [74]. GPR109A is expressed in a wide variety of tissues/cells including adipose tissue, skin, hepatocytes, retinal cells, and bones; it is also highly expressed in immune cells such as macrophages and dendritic cells [90,91]. Butyrate is the only known SCFA agonist for GPR109A, but it activates the receptor only with low affinity (*EC*_50_ value in low millimolar range) [72,73]. Acetate and propionate do not interact with the receptor. The receptor is expressed in the intestinal tract, most robustly in the colon where butyrate is present at concentrations high enough to activate the receptor [72,73]. The expression is restricted to the lumen-facing apical membrane of intestinal and colonic epithelial cells, thus having direct access to butyrate in the lumen. As such, the bacterial metabolite butyrate appears to be the principal agonist for GPR109A in the intestinal tract. Colonic bacteria regulate the expression of the receptor in the colon; the expression is markedly reduced in germ-free mice, but the expression comes back to normal when the colon gets re-colonized [73].

Upon ligand binding, GPR109A couples through the Gi pathway in most tissues, resulting in decreased levels of cAMP inside the cells [87,88]. The receptor functions as a suppressor of inflammation and carcinogenesis in the colon [72,92,93]. Deletion of the receptor in mice accelerates the progression of colonic inflammation and colon cancer in multiple experimental model systems [92,93]. The receptor is also expressed on mucosal immune cells, particularly in dendritic cells. Activation of the receptor in dendritic cells promotes the ability of these cells to convert naïve T cells into immunosuppressive Tregs and potentiates the production of the anti-inflammatory cytokine IL-10; Gpr109a-null mice have reduced number of Tregs in the colon, reduced levels of IL-10, and increased levels of the pro-inflammatory cytokine IL-17 [92,93]. Studies with NLRP6 knockout mice have shown that increased Prevotellaceae and decreased IL-18 levels are associated with colitis [94]. Gpr109a-null mice also show decreased IL-18 production and increased population of Prevotellaceae, both alterations leading to potentiation of experimentally induced colitis [92]. Recent studies show that NLRP3-mediated inflammasome plays an important role in IL-18 secretion and regulation of microbiota through GPR109A [93].

## 8. GPR43: Expression and Function in the Colon and the Mucosal Immune System

GPR43 was discovered in a cluster of four novel GPRs located in close proximity to CD22 on human chromosome 19 [95] and then cloned from mouse leukemia cells [96]. It has a widespread expression pattern, with most prominent expression in the adipose tissue, gastrointestinal tract, leukocytes and neutrophils [75]. The receptor is coupled to both Gq and Gi/o, leading to a decrease in cAMP levels and the activation of phospholipase-C [50,75]. Studies with reporter mice have shown that Gpr43 is highly expressed not only in colonic epithelial cells but also in immune cells present in the colon lamina propria [76]. The receptor is activated by all three major SCFAs, acetate, propionate, and butyrate [77], which is in contrast to GPR109A that is activated solely by butyrate. 

The exact biological role of GPR43 under normal physiological conditions remains controversial with regard to whether the receptor suppresses or promotes inflammation and carcinogenesis in the colon. Several studies have demonstrated that the receptor plays a role in promoting inflammation and cancer [77,97,98,99]. GPR43 is expressed at higher levels in colorectal and gastric cancers and overexpression of the receptor in cancer cells potentiates their growth when xenografted in nude mice [97]. The receptor has also been shown to induce neutrophil chemotaxis and promote inflammation [77,98,99]. However, there are other reports that show the opposite; the receptor functions as a suppressor of colonic inflammation and carcinogenesis [55,100,101]. Tang et al. [100] have reported that GPR43 is silenced in colon cancer, both at the primary site and in metastatic spreads. Most colon cancer cells do not express the receptor, and when engineered to express the receptor ectopically, the cells undergo apoptosis upon exposure to the receptor agonists propionate or butyrate. These studies strongly demonstrate a tumor-suppressive function for the receptor. Maslowski et al. [55] and Masui et al. [101] have shown that Gpr43- null mice are more susceptible to experimental colitis, indicating an anti-inflammatory role for the receptor. Our own studies [102] also corroborated the function of the receptor as a suppressor of inflammation and carcinogenesis. The anti-inflammatory role is further supported by the findings that Gpr43 blocks inflammasomes [93] and deletion of the receptor in mice leads to dysbiosis in colonic microflora and increased susceptibility to colon cancer [102]. 

In contrast to GPR109A, which is expressed only in colonic epithelial cells, GPR43 is expressed in epithelial cells and in enteroendocrine cells, particularly L-cells [103,104,105]. This has significant biological implications. L-cells secrete two important gut hormones: glucagon-like peptide 1 and Peptide YY. Activation of GPR43 by SCFAs increases the secretion of both these gut hormones. As these hormones impact on the function of pancreas (insulin secretion) and brain (appetite control), SCFAs influence the biology of distant organs via GPR43 expressed in the intestinal tract. 

## 9. GPR41: Expression and Function in the Colon 

GPR41 is also a receptor for SCFAs: all three SCFAs activate the receptor [50]. However, similar to GPR43, GPR41 is expressed in enteroendocrine cells, but unlike GPR43, GPR41 is not expressed in colonic epithelial cells [104,105,106]. Furthermore, unlike GPR109A and GPR43, GPR41 is also in enteric neurons [107]. However, the intracellular signaling mechanism seems to be similar for all three SCFA receptors, which involve a decrease intracellular levels of cAMP in response to receptor activation. Activation of GPR41 by SCFAs present in the colonic lumen is capable of altering secretion of gut hormones to impact biology at distant organs. Studies have shown that activation of Gpr41 by SCFAs induces secretion of glucagon-like peptide 1 and Peptide YY to alter insulin secretion and appetite control [104,105].

## 10. GPR81 and GPR91: Expression and Function in the Colon

GPR81 is a cell-surface, G-protein-coupled receptor for the ubiquitous metabolite lactate [78] whereas GPR91, also present on cell-surface, is a G-protein-coupled receptor for the citric-acid cycle intermediate succinate [79]. Relatively less is known about the role of these receptors in the colon. Both these receptors are expressed in colonic epithelial cells and are likely to play a role in colonic biology because lactate is a major metabolite generated by normal colonic bacteria, particularly by Lactobacilli, and succinate is released by dead bacterial cells. The presence of these receptors in the colon and their ligands in the colonic lumen strongly suggest a potential connection between these receptors and colonic health. Further investigations are needed to understand the biological significance of these two receptors and their role in the communication between colonic bacteria and the host. 

## 11. Nuclear Receptors AhR, PXR, and FXR and Their Modulation by Bacterial Metabolites

There are bacterial metabolites other than the SCFAs that are also important for the communication between the host and colonic bacteria and hence for optimal colonic health. These include tryptophan metabolites and bile acids (Table 1). While the SCFAs elicit their biological effects on the colon by serving as agonists for the cell-surface G-protein-coupled receptors GPR109A, GPR43, and GPR41, tryptophan metabolites and bile acids impact colonic health by serving as the ligands for the nuclear receptors AhR (aryl hydrocarbon receptor), PXR (pregnane X receptor), and FXR (farnesoid X receptor). Unlike the G-protein-coupled receptors, which regulate cellular function via changes in intracellular levels of the second messengers such as cAMP and calcium, the nuclear receptors modulate cellular function by altering gene transcription as they all function as transcription factors. However, the ability of these nuclear receptors to bind to their target genes is ligand-dependent; the receptors reside in the cytoplasm when not bound to their ligands, but in the presence of the ligands, the receptor-ligand complex translocates into the nucleus to act on the target genes. 

AhR is expressed in colonic epithelial cells and functions as a suppressor of inflammation and carcinogenesis [62,108,109]. The bacterial metabolites that activate this receptor originate from tryptophan catabolism; these include tryptamine, indole, indole-3-aldehyde, indole-3-acetic acid, and indole-3-propionic acid (Table 1). Activation of AhR by these metabolites occurs at doses that are relevant to their concentrations found in the colonic lumen under normal physiological conditions [60,61]. AhR has received considerable attention for its activation by xenobiotics as a process related to xenobiotic biotransformation and their subsequent elimination from the body. However, recent studies have identified the endogenous tryptophan metabolite kynurenine as the physiological agonist for the receptor [81]. Kynurenine is a catabolic product of tryptophan in mammalian cells, generated by the intracellular enzyme indoleamine-2,3-dioxygenase-1 (IDO1); this enzyme is expressed at high levels in colonic epithelial cells. IDO1 is an immunosuppressive enzyme [110] and its robust expression in the colon contributes to the tolerance of the colonic bacteria by the host. Activation of AhR by kynurenine in the colon is obviously essential for the host-bacteria symbiotic relationship. The tryptophan metabolites produced by the colonic bacteria also contribute to this process via their ability to activate the same receptor. A more recent study has identified another tryptophan metabolite, indole-3-acrylic acid, in colonic lumen that is generated by a specific bacterial species, Peptostreptococcus [80]. This metabolite is an agonist for AhR and is also a potent activator of antioxidant machinery in cells [80]. Through these mechanisms, indole-3-acrylic acid promotes intestinal barrier function and attenuates inflammation.

PXR and FXR are also nuclear receptors and they are also activated by various xenobiotics. However, the endogenous agonists for these receptors are steroids and bile acids, respectively (Table 1). The bacterial metabolites that activate these receptors include the secondary bile acids deoxycholic acid and lithocholic acid and the tryptophan metabolite indole-3-propionic acid. The activation of both these receptors elicits protective effects in the colon against inflammation and cancer [82,83,84,85,86].

## 12. Relevance of Dietary Fiber to the Production of Tryptophan Metabolites in Colon

Colonic bacteria generate tryptophan metabolites via protein metabolism. In contrast, the short-chain fatty acids are generated via fermentation of carbohydrates that reach the colon mostly in the form of dietary fiber. However, the quantity and quality of dietary fiber can potentially impact the generation of tryptophan metabolites because fiber as the source of carbon and energy is a major determinant of the composition of bacterial species in the colon. It has been documented that restriction of sugar in the diet promotes expansion of Lactobacilli species that possess enzymatic machinery to generate selective tryptophan metabolites to activate AhR and stimulate IL22 production [14]. This cytokine alters mucosal immune response in such a manner that it facilitates colonization of selective bacterial species and at the same time prevents colonization of the fungus Candida albicans [14]. As such, alterations in dietary fiber intake have a major impact on the generation of tryptophan metabolites and possibly other metabolites as well.

## 13. Conclusions

The symbiotic relationship between colonic bacteria and the host is undeniable, and this mutually beneficial coexistence is made possible through effective communication between the two partners in this co-habitation. The primary mode of this communication is chemical, mediated by specific bacterial metabolites that elicit their biological effects on the host by activating selective molecular targets in the host colon. Some of these molecular targets (GPR41, GPR43, GPR109A, GPR81, and GPR91) are located on the lumen-facing apical membrane of colonic epithelial cells where they have direct access to bacterial metabolites generated in the lumen. Some are located intracellularly (e.g., HDACs, AhR, FXR, PXR, and PPARs), and there is evidence that the bacterial metabolites generated in the lumen enter the colonic epithelial cells, many of them via selective transporters expressed in the apical membrane of these cells. Mucosal immune cells present in the lamina propria also express the same molecular targets as in colonic epithelial cells for these bacterial metabolites, but it is questionable if all of these metabolites reach lamina propria at concentrations sufficient to act on these targets in immune cells. This question seems particularly appropriate for SCFAs because these metabolites are actively metabolized in colonic epithelial cells. This might not be an issue for tryptophan metabolites. Taken collectively, there is an overwhelming evidence for active dialogue between colonic bacteria and the host with selective bacterial metabolites functioning as messengers. The end result of this chemical communication is the tolerance of colonic bacteria by the host immune system, which is obligatory for the symbiosis. A beneficial byproduct of this co-habitation and symbiosis is the maintenance of optimal colonic health with a decreased risk of colonic inflammation and carcinogenesis.

## Figures and Tables

**Table 1:** Receptors involved in host-bacteria communication in the colon.

Receptor	Agonist	
	Bacterial Metabolite	Endogenous Metabolite
**Cell-surface receptors**		
GPR109A	Butyrate [72,73]	β-Hydroxybutyrate [74]
GPR43	Acetate, Propionate, Butyrate [75,76,77]	Acetate, Propionate [75,76,77]
GPR41	Acetate, Propionate, Butyrate [75]	Acetate, Propionate [75]
GPR81	Lactate [78]	Lactate [78]
GPR91	Succinate [79]	Succinate [79]
**Nuclear receptors**		
AhR	Tryptamine, Indole, Indole-3-aldehyde, Indole-3-acetic acid, Indole-3-propionic acid [60,61], Indole-3-acrylic acid [80]	Kynurenine [81]
PXR	Lithocholic acid, Indole-3-propionic acid [82]	Steroids [82]
FXR	Deoxycholic acid, Lithocholic acid [83,84,85,86]	Cholic acid, Chenodeoxycholic acid [83,84,85,86]
